# Practical Departments

**Published:** 1905-08-12

**Authors:** 


### PRACTICAL DEPARTMENTS.

### THE BRUUN-LOWENER WATER SOFTENER.

All forms of water softener are designed to accomplish the same object, the elimination of the substances that are present in the water and that will be deposited in the working of the boiler, the calorifier, or other heating apparatus, upon the surfaces of the metal, through which the heat is to pass to the water, or their conversion into substances that are not deposited, and the different apparatus represent the views of the various inventors. In the Bruun-Lowener, the whole of the water that is to be used for raising steam, for heating, or for laundry work, is submitted to a process that, it is claimed, eliminates both the temporary hardness and the permanent hardness at one operation. It will be remembered that by temporary hardness is meant the presence of certain bi-carbonates, which are soluble in cold water, but which when the water is heated and a portion of the carbonic acid present is driven off, become carbonates, and are deposited, while by permanent hardness is meant the presence of certain sulphates and chlorides, which are not precipitated by boiling, and for which chemical reagents have to be employed. In the Bruun-Lowener apparatus all the water is boiled and filtered after boiling, so that all the carbonates are removed, and in addition all the water entering the apparatus is mixed with a certain definite quantity of chemical reagents, determined by the quantity of the sulphates and chlorides that are present, and the whole arrangement is automatic, once the hardness is known and the plant fixed.

#### The Apparatus.

The apparatus consists of an outer receptacle, which takes various forms, according to the quantity of water to be dealt with, and the conditions of the service. Favourite forms are one of rectangular section and one cylindrical. The receptacle contains a receiving chamber, which also acts as a mixing chamber for the chemicals and the water, a heating chamber, a depositing chamber, and a filter. Above the receiving chamber a measuring receptacle is fixed, consisting of two tanks arranged to oscillate on a pivot. One of the tanks is always under the water supply pipe, which is brought to a convenient position, and when it is full it tumbles over, in the manner well known, emptying its load of water into the receiving chamber below, and bringing the other tank under the supply pipe. Close to the oscillating tanks is a semi-cylindrical tank containing the chemical reagents, a certain definite quantity of which passes from the chemical tank into the receiver below, each time the measuring tank tumbles over, a system of levers attached to the oscillating tanks actuating a valve in the bottom of the tank containing the chemicals. The chemicals in the semi-cylindrical tank are kept in motion by a stirrer also worked by the oscillating motion of the tumbling tanks. The chemicals, principally lime milk, in the chemical tank are in a very concentrated state so that a small quantity added to the water is sufficient to accomplish the neutralising of the sulphates and chlorides. The mixture of water and chemicals passes from the receiving chamber to the heating chamber where it is heated by means of steam passing in a coil of pipe. It then passes downwards into the depositing tank, which has a large capacity, where the carbonates that have been separated out by heating, and the other insoluble salts that have been formed by chemical action, are deposited on the bottom, and drawn off from time to time by a sludge cock. From the depositing chamber, the water passes up through the filter, which occupies the top of the chamber, so that all the water must pass through it on its way out. The filter consists of wood wool, packed between two rows of wooden bars. After passing through the filter, the water runs into the storage tank arranged to receive it at the side. The filter can be cleaned as often as required by removing the top wooden bars and taking out the wood wool, the wool being used over and over again after cleansing. A Bruun-Lowener water-softener is in use at the Middlesex Hospital, capable of dealing with 600 gallons an hour. It softens the water and raises the feed-water to a temperature of 190° F. Before the water-softener was put in the boilers required cleaning every month, and it then took two men eight days to clean them, now they can run two months, and one man can clean them in two days. The English agents are Messrs. Lassen and Hjort, of 52 Queen Victoria Street, London, E.C.

